# Isolating Spermathecae and Determining Mating Status of *Drosophila suzukii*: A Protocol for Tissue Dissection and Its Applications

**DOI:** 10.3390/insects8010032

**Published:** 2017-03-10

**Authors:** Alina Avanesyan, Benjamin D. Jaffe, Christelle Guédot

**Affiliations:** Department of Entomology, University of Wisconsin-Madison, 1630 Linden Drive Madison, WI 53706, USA; bjaffe2@wisc.edu (B.D.J.); guedot@wisc.edu (C.G.)

**Keywords:** *Drosophila suzukii*, invasive species, mating status, spermathecae, sperm storage, spotted wing drosophila, tissue dissection

## Abstract

The spotted wing drosophila, *Drosophila suzukii* (Diptera: Drosophilidae), is an emerging invasive pest, which attacks a wide variety of fruits and berries. Although previous studies have focused on different aspects of *D. suzukii* reproductive biology, there are no protocols available for determining the mating status of *D. suzukii* females and drosophilids in general. In this study, a step-by-step protocol for tissue dissection, isolating spermathecae, and determining the mating status of females was developed specifically for *D. suzukii*. This protocol is an effective and relatively quick method for determining female mating status. It has important applications from exploring reproductive output of *D. suzukii* females to understanding the biology of *D. suzukii* winter morph, which presumably plays the main role in the overwintering of this invasive species. We demonstrated applicability of this protocol for both field collected flies and flies reared in the lab, including fly specimens stored on a long-term basis.

## 1. Introduction

The spotted wing drosophila, *Drosophila suzukii* Matsumura (Diptera: Drosophilidae), native to Southeast Asia, is an emerging invasive pest in Europe, North America, and South America, which attacks a wide variety of soft-skinned fruits and berries [1,2,3,4,5]. First detected in Hawaii in 1980s, *D*. *suzukii* acquired a pest status in the United States only in 2008 after its detection in California [3,4].

Although the host preferences and phenology of *D. suzukii* have been extensively explored [3,6,7,8,9,10], the reproductive biology of *D. suzukii* has received less attention in entomological studies. The few studies of the sexual behavior of *D. suzukii* explored female oviposition, male courting behavior, and the production of sex pheromones [11,12,13]; however, the morphology and development of the reproductive organs in *D. suzukii* in relation to female fertility and mating behavior are still poorly understood. Meanwhile, understanding the reproductive biology of *D. suzukii* is important for effective control of this invasive species [13].

One of the important steps towards exploring *D. suzukii* reproductive biology and ecology is to accurately determine a female’s mating status. It has been demonstrated with several fruit fly species that mating can induce different physiological changes in females such as: (1) tissue differentiation in the oviduct, which may result in increased egg production [14,15]; (2) strong rejection of males after mating [14]; and (3) changes in female longevity [16]. As *D. suzukii* is such a detrimental invasive species, it is also important to know female mating status at different times during a year to better understand *D. suzukii* seasonal phenology. Particularly, such information can be helpful for studying *D. suzukii* reproductive diapause; that was suggested by Asplen et al. [4] as one of the important directions for studies involving this species.

The mating status of *D. suzukii* females can be determined by detecting the presence of sperm in one of two sperm storage organs: (1) the paired spermathecae (long-term sperm storage) connected with the paired spermathecal glands or (2) a single seminal receptacle (short-term sperm storage) [17,18]. As in any other drosophilid species, after mating, a *D. suzukii* female stores sperm received from a male and releases it later to fertilize mature eggs. Although the seminal receptacle could serve as the principal sperm storage organ [19] and may store a larger amount of sperm than the spermathecae, the sperm from the seminal receptacle releases sooner than that from the spermathecae [17,20,21]; consequently, detecting the sperm in the spermathecae rather than in the seminal receptacle could potentially confirm that the female has mated.

**Figure 1:**
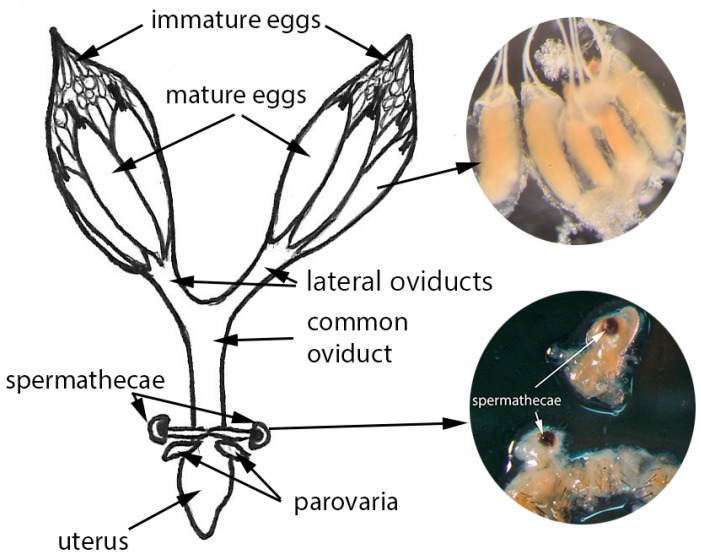
Scheme of *Drosophila suzukii*‘s reproductive system from the dorsal perspective; the seminal receptacle is not shown due to its location on the ventral side of the common oviduct. (The drawing is by Claire Mattmiller; images by Kathryn Hietala-Henschell and Alina Avanesyan).

The spermathecae are paired structures; each of them consists of a spermathecal reservoir and a spermathecal duct, which connects the spermatheca with the common oviduct (Figure 1).

It was demonstrated with *D. melanogaster* that, during mating, sperm is transferred from the uterus to the spermathecal reservoir and coils around the reservoir’s ‘center’ forming a toroidal mass [17]; the spermathecae can contain the sperm from multiple males and store it for up to two weeks [20]. The toroidal mass can be easily detected under the light microscope; consequently, its presence or absence can be used as a criterion for determining a female’s mating status.

Spermathecae morphology has been studied in several orders; Blattodea [22], Orthoptera [23], Coleoptera [24,25,26,27], Heteroptera [28,29,30,31], Hymenoptera [32,33], and Diptera [34,35,36,37,38,39] including *D. melanogaster* [17,18,19,21]. However, to the best of our knowledge, there is only one recent study on *D. suzukii* spermathecae morphology [40], and there are no studies on determining *D. suzukii* mating status. Some of the previous studies focusing on *D. suzukii* phenology and its attraction to different bait mixtures involved dissections of the fly reproductive tract and reported the mating status of flies based on sperm presence in the spermathecae [41]. However, these studies provided neither details on how the spermathecae were isolated and how the sperm mass was detected nor images of a sperm-containing spermatheca, which could be used as a reference for future research on *D. suzukii* reproductive biology. In addition, different studies can use different fly specimens (flies reared in lab or field collected and preserved flies); consequently, it is important to know whether determining the mating status by isolating the spermathecae can be effective for different fly specimens.

To address these issues, in this study we (1) provide a detailed protocol for *D. suzukii* tissue dissection, isolating spermathecae and determining a female’s mating status, and (2) demonstrate the applicability of this protocol for flies reared in the laboratory and for preserved field-collected specimens.

## 2. Materials and Methods

### 2.1. Study Species

For the protocol development, *D*. *suzukii* individuals from a laboratory colony housed at the University of Wisconsin-Madison were used; the flies were originally collected in 2015 from infested raspberries in Wisconsin. The fly stock was maintained at room temperature (at around 25 °C) on a standard molasses-based diet containing 4500 mL water, 500 g cornmeal, 500 g molasses, 200 g yeast, 54 g agar, 20 mL 100% propionic acid, and 45 mL 20% tegosept in 95% ethanol (provided by the Department of Genetics, University of Wisconsin-Madison). In April 2016, ten females were randomly selected from the rearing vials and collected within two hours after hatching (presumably virgin); similarly, ten females were randomly selected and collected 24 h after hatching or later (presumably mated). Virgin and mated females were transferred to new vials and were stored separately in 70% ethanol at room temperature until they were dissected.

### 2.2. Protocol Development

#### 2.2.1. Step 1: Dissection and Isolation of Spermathecae

**Figure 2:**
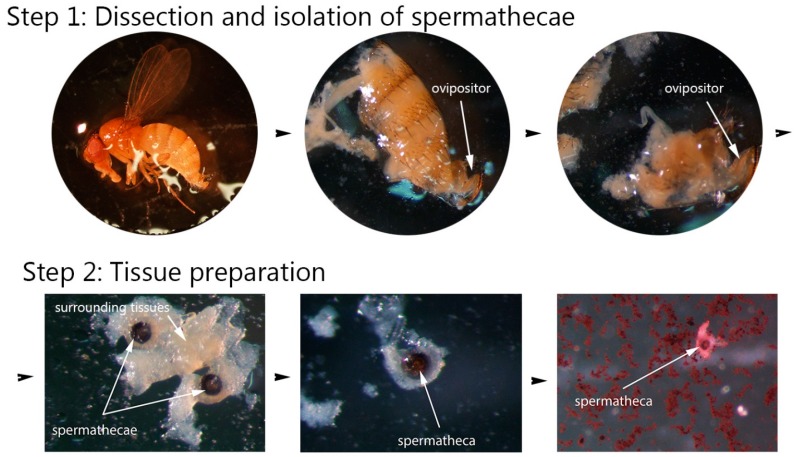
Basic steps of dissecting female *Drosophila suzukii* flies and isolating spermathecae. Step 1 includes cutting the exoskeleton of the abdomen and pulling out the reproductive system; step 2 includes removal of the spermathecal glands and surrounding tissues and staining the spermathecae (Images by Alina Avanesyan).

Each female fly from each subsample (virgin and mated) was placed in a Petri dish in a drop of distilled water (Figure 2). Under the dissecting microscope (OLYMPUS SZX16, Olympus America Inc., Center Valley, PA, USA), the abdomen of the fly was open using a pair of fine tweezers from the micro dissecting kit (BioQuip Products Inc., Rancho Dominguez, CA, USA; micro dissecting kit, Cat. No. 4761). Then the ovipositor with the spermathecae and spermathecal glands were pulled out, transferred to a microscope slide, and placed in a drop of water (Figure 2). On the slide, both spermathecae were separated from the rest of the tissues using the micro slide tool kit (BioQuip Products Inc., Rancho Dominguez, CA, USA; micro slide tool kit, Cat. No. 4831). Each dissection step, as well as all the slides with isolated spermathecae, were photographed with an Olympus DP73 digital camera using the cellSens software package (Olympus).

#### 2.2.2. Step 2: Tissue Preparation

The spermathecal glands were removed as much as possible to make staining and further observation of the spermathecae and sperm mass easier (Figure 2). The ovipositor and all remaining tissues were also removed from the slide before staining. A small drop of 2% aceto-orcein (Thermo Fisher Scientific Inc., Pittsburgh, PA, USA) was added to the water drop with the spermathecae, and the slide was immediately covered with a cover slip.

#### 2.2.3. Step 3: Determining Mating Status

**Figure 3:**
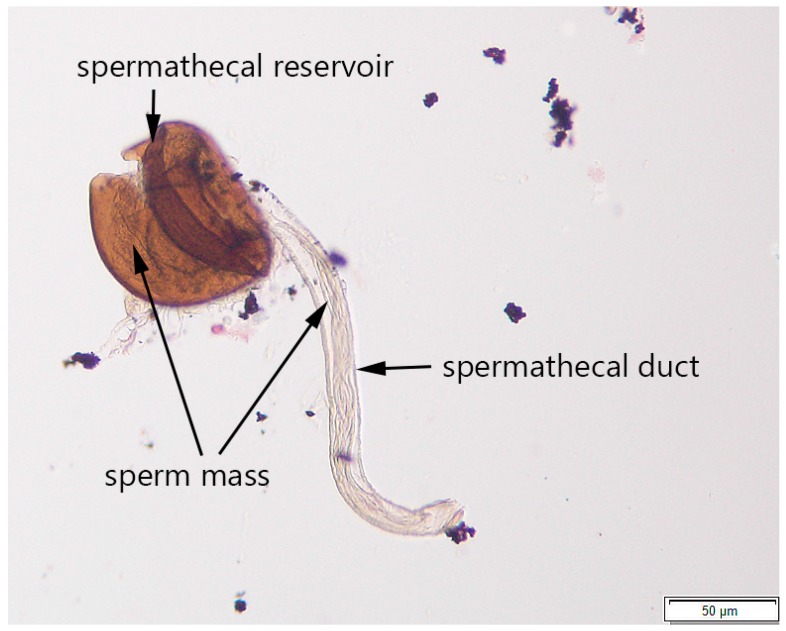
A spermatheca of a mated *Drosophila suzukii* female under the compound microscope at 20× objective lens; the scale is 50 µm (Image by Claire Mattmiller).

The content of the intact spermathecae and their surrounding tissues (if not all the tissues had been successfully removed) was observed under the compound microscope (Wild M20, Wild Heerbrugg, Heerbrugg, Switzerland) using 25× and 40× objective lenses. The mating status of the fly was tentatively determined as virgin if no toroidal mass was present in the spermathecae and the walls of the spermathecal reservoir and the spermathecal gland were uniform in appearance. If the spermathecal reservoir contained a toroidal mass (distinguishable under both 25× and 40× objective lenses), the fly was described as mated. The spermatheca was then photographed with an Olympus DP73 (using 20× and 40× objective lenses) for future reference (Figure 3).

**Figure 4:**
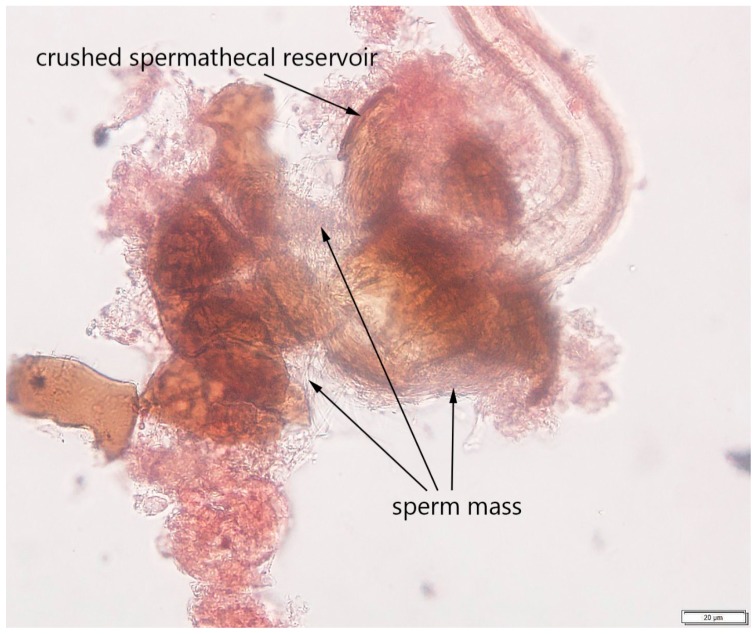
A crushed spermatheca of a mated *Drosophila suzukii* female observed under the compound microscope at 40× objective lens. The toroidal sperm mass is indicated with arrows; the scale is 20 µm (Image by Alina Avanesyan).

Due to the different appearance of the stained spermathecae under the microscope (e.g. the walls of the spermathecal reservoir could occasionally be broken, the tissues could be overstained, etc.) determining a fly’s mating status could be challenging. To confirm the presence or absence of the sperm mass in our study, the spermathecae were gently crushed on the slide under the cover slip using a pencil-top eraser and then repeatedly observed using 25× and 40× objective lenses. If the fly had mated, the released sperm mass was visible between broken parts of the spermathecal reservoir’s walls and in the surrounding area (Figure 4). If the fly was virgin, no sperm was observed after crushing the spermathecae.

### 2.3. Testing the Protocol

The applicability of the proposed protocol for flies other than those reared from the lab colony was demonstrated on (1) preserved field collected flies and (2) flies reared from fruit samples.

To determine the mating status of field collected flies, 48 female flies were randomly chosen from the preserved samples and dissected following the protocol’s steps. The flies were collected from infested raspberries at three different locations in Wisconsin in 2014 and had been preserved in 70% ethanol in the laboratory, as described in Pelton et al. [10]. Before the collection, the flies remained immersed in the yeast-sugar bait within the traps for one week.

To determine the mating status of flies reared from fruit samples, 50 late stage pupae were removed from another lab colony (the flies were originally collected near Fennville, MI, USA in 2015) and placed in individual 1 oz polyethylene containers with lids. The containers with the pupae were then placed in a growth chamber under a 16:8 L:D light cycle at 26 °C and monitored every day for the emergence of adults. Newly emerged adults were sexed within 24 h. Then, twenty females were randomly selected and placed individually in 1 oz containers containing fruit (2–4 g of raspberry). The females were then randomly arranged in two groups: (1) ten females were isolated into individual containers (‘virgin’ treatment) and (2) ten females had two males introduced into the containers (‘mated’ treatment). The lid of each container was perforated with three small holes (1mm); and a 2 cm^2^ square piece of filter paper (Fisherbrand^TM^ P8, purchased from Thermo Fisher Scientific Inc., Pittsburgh, PA, USA) was placed underneath the raspberry. A separate set of ten individual cups, each with 2–4 g of raspberry, was similarly prepared to ensure no prior infestation of raspberries; no flies were added to those cups. All the cups (with and without flies) were placed for 72 h in the growth chamber under a 16:8 L:D light cycle, at 23 °C. After 72 h, the flies were immobilized using CO_2_ and the females were placed into 95% ethanol. After the adults were removed, the raspberries were checked for the presence of eggs daily for three consecutive days or until the eggs were first observed. Starting on day 3, the raspberries were checked for the presence of larvae for five days or until the larvae were first observed.

Each female was then assigned a code, and a blind assessment of the mating status of each fly was conducted using the developed protocol for isolating the spermathecae (described above). The numbers of mated and unmated flies were recorded, and the numbers of correct identifications were concluded by comparing the identification results with the type of the treatment (‘virgin’ and ‘mated’). The Kruskal-Wallis test was then used to determine whether the numbers of correct identifications differed between the treatments.

## 3. Results

### 3.1. Protocol Development

The protocol for isolating the spermathecae and determining fly mating status was developed using ten presumably virgin and ten mated females. The spermathecae of 17 dissected females were isolated and were screened under the compound microscope for the presence or absence of sperm. The spermathecae containing sperm mass (Figure 3 and Figure 4), as well as the spermathecae without sperm mass (image is not provided), were photographed; these images then served as references for subsequent testing of the protocol on different fly specimens. Due to considerable sperm length in drosophilids, the sperm mass was easily distinguishable in the spermathecae [42]. The results showed that all ten females dissected within two hours after emergence were characterized by the absence of sperm in both spermathecae, while seven out of ten females collected 24 h after emergence or later contained sperm. We were not able to determine the presence or absence of sperm in spermathecae from three other presumably mated females due to our inability to locate the spermathecae on the slide after covering it with the cover slip or due to overstaining the slide.

### 3.2. Testing the Protocol

**Figure 5:**
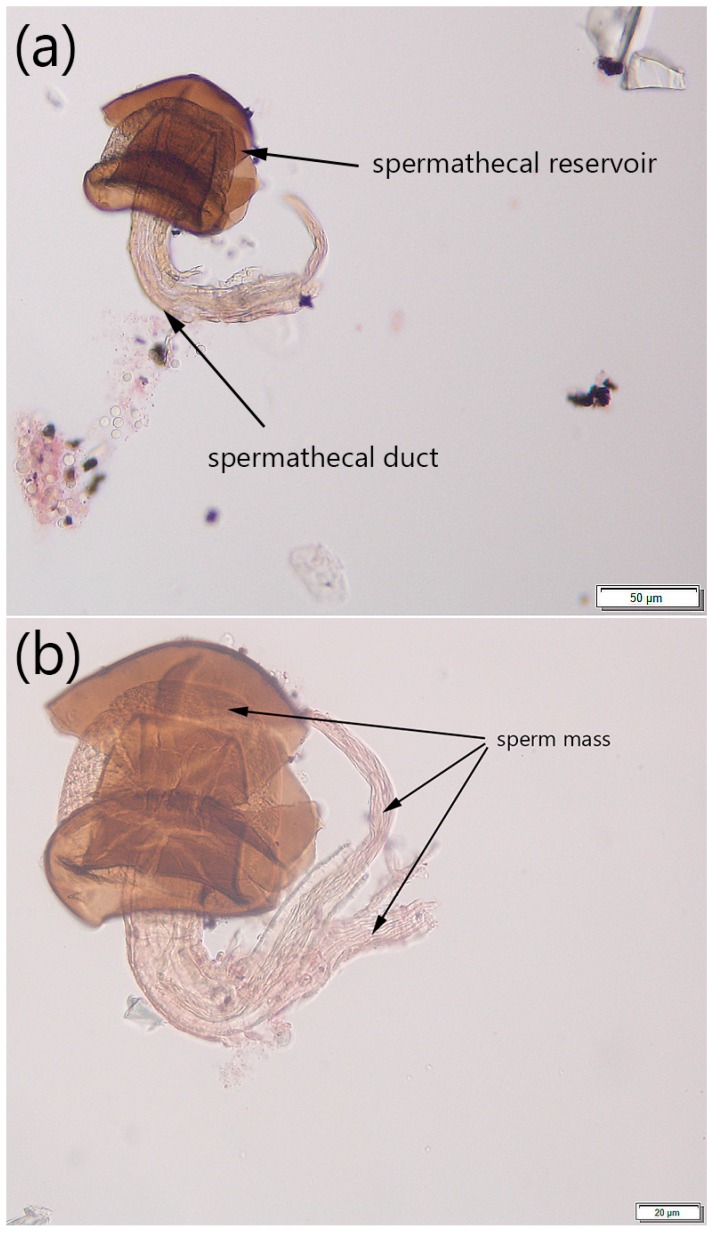
Spermathecae isolated from field collected *Drosophila suzukii* females. (**a**) Intact spermatheca (at 20× objective lens; the scale is 50 µm); (**b**) Crushed spermathecae with toroidal sperm mass indicated (at 40× objective lens; the scale is 20 µm) (Images by Claire Mattmiller).

The proposed protocol was successfully applied for about 90% of field collected female flies (Figure 5 and Table 1). In five out of 48 flies we were unable to determine the female’s mating status due to dissection and staining issues, i.e., in three flies the spermathecae were ‘lost’ during the dissection or transferring the slide to the compound microscope; one slide was overstained and the sperm in the spermathecae was difficult to observe, and the spermathecae on one slide were ‘overcrushed’.

The appearance of the spermathecae in the field collected flies slightly differed from that in the flies from the lab colony; in most cases, the walls of the spermathecal reservoir were ‘wrinkled’ and easy to crush, and the sperm was visible in the surrounding tissues even before crushing (Figure 5).

The spermathecae were successfully isolated from 100% of the flies reared from the berries in the lab (Table 2). The appearance of the spermathecae in mated females was similar to that of the field collected flies (Figure 5). The larvae were observed in all the vials with the ‘mated’ treatment confirming that all the dissected females from these vials were indeed mated. The larvae were not observed in the vials with ‘virgin’ treatment, as well as in the control vials with raspberry only; this confirmed that the dissected females from these vials were unmated and that raspberry was not previously infected.

The mating status was correctly determined for 60% females from the ‘virgin’ treatment and 70% females from the ‘mated’ treatment; 65% of the total number of flies were correctly identified.

We did not find a significant difference between the numbers of females with correctly determined mating status in the ‘virgin’ and the ‘mated’ treatments (Kruskal-Wallis test: *H* = 0.2, df = 1, *p >* 0.05).

## 4. Discussion

Accurately determining the mating status of *D. suzukii* females is a necessary step in studies on reproductive biology and seasonal phenology that involve dissecting flies [15]. Considering that this species is highly invasive [4], the availability of an effective protocol for tissue dissection and detection of sperm mass in the spermathecae can be essential for understanding *D. suzukii* biology and developing effective control strategies. To the best of our knowledge, this is the first attempt to develop a step-by-step protocol for isolating spermathecae and determining the mating status of the invasive *D. suzukii*. We also obtained images of the spermathecae from mated flies, which could be used as a reference for future studies involving fly tissue dissection and determining a female’s mating status. In addition, we demonstrated the effectiveness of the developed protocol for both ‘fresh’ fly individuals reared in the lab and fly specimens collected from the field and preserved in ethanol for about two years.

The results from the protocol validation using flies reared from raspberry in the lab suggested that successful determination of a female’s mating status might depend on accurate dissection and tissue preparation. We were unable to correctly determine the mating status in 35% of females due to the following: (a) the presence of some other fly tissues and organs on the slide, which often overlapped with some parts of the spermathecae; (b) a lack of some parts of the isolated spermathecae; and (c) squashing, overstaining, or overcrushing of the spermathecae. The lack of difference between the numbers of correctly identified flies from the ‘virgin’ and ‘mated’ treatments also suggests that careful tissue preparations could be critical and, once it is done accurately, detecting the sperm mass within the spermathecal reservoir or even spermathecal duct (Figure 3 and Figure 5) could be performed without issues. Additionally, the dissections for developing this protocol were conducted in water, which might explain our issues with tissue preparations. Following other studies that involved tissue dissections in *Drosophila* [43,44] we recommend using a standard saline to minimize osmotic damage to the tissues.

The described protocol has many important applications for studies on *D. suzukii* and on both other drosophilids and non-drosophilid flies. For example, considering the seasonal variation in *D. suzukii*, which is currently extensively explored [45,46], information about the mating status of *D. suzukii* winter morphs would allow us to better understand the reproductive state in which *D. suzukii* females can potentially overwinter. Applicability of the protocol for different fly specimens is especially useful for studies on *D. suzukii* seasonal phenology when researchers might not be able to process the fly trap catches right after collecting, or when data from multiple years are analyzed. In addition, it has been demonstrated on tephritids that sperm presence in the spermathecae can help accurately differentiate sterile females from fertile ones, especially when other methods such as using fluorescent dye may fail [39]. Finally, the developed protocol is a relatively quick method for determining a fly’s mating status; it takes approximately 15–20 min to dissect one fly, isolate the spermathecae, and detect the presence of the sperm mass.

While developing this protocol, in addition to possible issues with fly dissection (described above), we identified the following potential difficulties: (1) occasional inability to locate the spermathecae on a slide due to its shifting during staining; (2) overstaining of the slide; and (3) overcrushing of the spermathecae. The first two issues can be addressed by extremely gentle staining of the slide with an aceto-orcein solution and observing the whole process of staining under the dissecting microscope. To prevent overstaining of the spermathecae, we recommend placing a drop of the stain on the slide at a 1 mm distance from the water drop; then, using a dissecting needle, the stain can be lightly mixed with water solution around the spermathecae. To prevent overcrushing of the spermathecae, we recommend crushing the spermathecae only when the presence of the toroidal mass is not clear (in most flies dissected in this study the toroidal mass was well distinguishable in an intact spermatheca even at 25×) or when only one spermatheca was isolated.

It is possible that detecting sperm within a seminal receptacle might also provide an accurate way to determine a female’s mating status. It can be especially applicable to those *Drosophila* species that could primarily use the seminal receptacle to store sperm [19] and rarely rely exclusively on the spermathecae. This could be addressed in future studies on the reproductive biology of *D. suzukii*.

Spermathecae play an important role in the reproduction of fly females: sperm storage increases both female fecundity and fertility while allowing females to save the energy needed for repeated matings [17,18]. The developed protocol is a helpful tool for detecting the presence of the sperm mass in the spermathecae, determining the reproductive status of fly females and predicting their reproductive behavior during a season. Following Revadi et al. [13], by developing this protocol we would like to stimulate research on the reproductive biology of *D. suzukii*, which may provide us with an important tool for the effective control of this highly invasive species.

## 5. Conclusions

In this study, we developed a protocol for determining a female’s mating status, which can be used in various studies on *D. suzukii* reproductive biology. We also demonstrated that this protocol could be used for both flies reared in lab and field-collected flies, as well as for flies preserved in ethanol for about two years. The developed protocol might have potential issues, and we provided suggestions for improving dissection and tissue preparation. Using this protocol will be helpful in studies on the reproductive biology of *D. suzukii* and especially in studies exploring the reproductive winter diapause of this invasive species.

## Figures and Tables

**Table 1:** Efficiency of the protocol for determining mating status in different *Drosophila suzukii* individuals collected across different locations in Wisconsin in 2014.

Preservation Time (Days)	Mating Status ^1^	
	Virgin	Mated
565	0	5
572	0	4
575	0	5
579	2	2
579	0	1
582	0	3
589	0	3
590	1	0
593	0	1
603	0	5
603	?	?
645	1	0
667	0	5
691	0	5

^1^ Undetermined females are denoted as ‘?’.

**Table 2:** Efficiency of the protocol for determining the mating status of *Drosophila suzukii* females reared in the laboratory from infested raspberries.

Parameters	Mating Status		Total Number of Flies
	Virgin	Mated	
Number of flies per treatment	10	10	20
Number of correctly identified flies	60%	70%	65%
Data comparisons (Correctly identified virgin vs. mated flies)	Kruskal-Wallis test: *H* = 0.2, df = 1, *p >* 0.05		
